# xsGastrointestinal symptoms are associated with a lower risk of hospitalization and mortality and Outcomes in COVID-19

**DOI:** 10.1186/s12876-022-02190-4

**Published:** 2022-03-10

**Authors:** Alireza Delavari, Samaneh Asgari, Yousef Alimohamadi, Abbass Vosoogh-Moghaddam, Anahita Sadeghi, Shokouh Shahrousvand, Armin Zakeri, Rahmatollah Moradzadeh, Samaneh Akbarpour

**Affiliations:** 1 Digestive Diseases Research Center, Digestive Diseases Research Institute, Shariati Hospital Tehran University of Medical Sciences, Tehran, Iran; 2 Prevention of Metabolic Disorders Research Center, Research Institute for Endocrine Sciences, Shahid Beheshti University of Medical Sciences, Tehran, Iran; 3 Health Research Center, Life Style Institute, Baqiyatallah University of Medical Sciences, Tehran, Iran; 4 Brain and Spinal Cord Injury Research Center, Neuroscience Institute, Tehran University of Medical Sciences, Tehran, Iran; 5 Department of Internal Medicine, School of Medicine, Shariati Hospital, Tehran University of Medical Sciences, Tehran, Iran; 6 Department of Epidemiology and Biostatistics, School of Public Health, Tehran University of Medical Sciences, Tehran, Iran; 7 Department of Hematology, Faculty of Medical Sciences, Tarbiat Modares University, Tehran, Iran; 8 Department of Epidemiology, School of Health, Arak University of Medical Sciences, Arak, Iran; 9 Sleep Breathing Disorders Research Center, Tehran University of Medical Sciences, Tehran, Islamic Republic of Iran; 10 Occupational Sleep Research Center, Baharloo Hospital, Tehran University of Medical Sciences, Tehran, Iran

**Keywords:** Gastrointestinal, Respiratory, Hospitalized, COVID-19, Symptoms

## Abstract

**Background:**

We aimed to find the association between gastrointestinal (GI) and respiratory symptoms with mortality and hospitalization among COVID-19 patients.

**Methods:**

We analyzed the registered data of COVID-19 patients from February 20, 2020, to March 10, 2021. Depending on the patients’ disease symptoms, four categories were defined: patients with only GI symptoms, patients with only respiratory symptoms, patients with both symptoms, and patients with other symptoms. Logistic regression analysis was used to assess the association of groups with outcomes.

**Results:**

A total of 42,964 patients from 23 hospitals were included, of which 26.5% patients had at least one or more GI symptoms. Of total patients, 51.58% patients were hospitalized among which 22.8% had at least one or more GI symptoms. GI symptoms significantly decreased the odds of mortality (OR 0.72, 95% CI 0.56–0.92), but respiratory symptoms increased the odds for mortality (1.36: 1.24–1.50), compared with patients with other symptoms. Moreover, the odds ratio of patients who had both respiratory and GI symptoms increased (1.52: 1.31–1.78) compared with patients with other symptoms. The same results were observed for hospitalization as the outcome.

**Conclusions:**

Our study showed that the presence of GI symptoms in COVID-19 at the time of admission was associated with a lower odds of hospitalization and mortality; however, this association had higher odds for respiratory symptoms.

## Introduction

In December 2019, pneumonia cases were first reported in Wuhan, China [1]. Despite efforts by national and international organizations worldwide to prevent the spread of acute coronavirus syndrome, COVID-19 disease has become a pandemic [2]. Research shows that the mortality rate of COVID-19 is estimated to be between 1.4 and 61% and the case fatality rate between 3.4 and 11% [3, 4].

Initially, the most common symptoms of COVID-19 were fever, cough, and shortness of breath. After a while, asymptomatic infections, chills, nasal congestion, fatigue, myalgia, sore throat, nausea, vomiting, diarrhea, and rhinorrhea were reported [5]. The COVID-19 virus causes acute respiratory distress syndrome (ARDS) by damaging the lungs' alveolar epithelial and endothelial cells. ARDS occurs due to a critical systemic inflammatory reaction that is more common than other organ injuries [6]. Some studies report that 75% of COVID-19 patients experience one or more gastrointestinal (GI) symptoms [7]. Diarrhea is the most common symptom in patients with GI infection symptoms, affecting 11.5% of patients. After that, nausea and vomiting (6.3%) and abdominal pain (2.3%) are common among these patients [8]. SARS-CoV-2 affects host cells through angiotensin-converting enzyme receptors (ACE2). In addition, pulmonary AT2 cells are expressed in the GI tract (cells of the esophagus, pancreas, hepatobiliary tree, small intestine, and large intestine) [9]. According to the "gut-lung axis," changes in the composition and function of the flora of the respiratory system through the immune system affect the GI system, and disorders of the flora of the GI tract through the standard mucosal immune system affect the respiratory system. Bacteria and toxins enter the bloodstream after intestinal mucosa and organs far from the body are damaged [10]. Although GI symptoms are usually associated with a poor prognosis in COVID-19 patients, they are very common in these patients [11, 12]. About 17.5% of GI patients are classified as severely ill [8]. A meta-analysis estimated odds of mortality in GI patients at 0.92% [13].

Approximately 5% of patients with COVID-19 and 20% of hospitalized patients experience severe symptoms that require intensive care. Also, more than 75% of hospitalized patients need extra oxygen [14]. The infection can lead to acute respiratory distress syndrome and even death [15]. Numerous studies have been conducted to identify prognosis or risk factors for death and severity in hospitalized patients with Covid-19. People over 60 years of age and patients with underlying diseases (including hypertension, diabetes, cardiovascular disease, chronic respiratory disease, and cancer), patients with respiratory symptoms and impaired clinical parameters (inflammatory markers, lymphopenia, and respiratory function) are at the highest risk for severe disease and death [2, 16]. Despite the high prevalence of gastrointestinal symptoms, there is limited information on the adverse outcomes of patients with GI symptoms in tall sample sizes [17]. Limited studies have also examined outcomes in patients with both GI and respiratory symptoms. Accordingly, this study aimed to compare the mortality and hospitalization among patients with various GI and respiratory clinical symptoms to identify patients at risk of death and apply appropriate interventions.

## Methods

### Study population

The present study was a secondary analysis of the data from 14 hospitals (Ziaeian, Amir Alam, Arash, Baharloo, Bahrami, Farabi, Sina, Tehran Heart center, Imam Khomeini, Razi, Roozbeh, Shariati, Yas, and Children's Medical Center) affiliated with the Tehran University of Medical Sciences and health services (TUMS) as referral hospitals for COVID-19; these hospitals provide about 30% of the care in the Tehran province.

We used the medical care monitoring center (MCMC) database for COVID-19 patients during the COVID-19 Epidemic in Tehran. MCMC database is a comprehensive and online system to register COVID-19 patients in hospitals. COVID-19 was confirmed by RT-PCR or was based on clinical symptoms of COVID-19 infection, including fever or respiratory symptoms with lung imaging features on chest CT consistent with the radiological criteria of COVID-9 infection [18], routinely utilized as a primary and sensitive tool for diagnosis of COVID-19 in our country [19, 20].

Because most mortality and hospitalization cases occurred among the people with ≥ 30 years, the study population included Iranian patients aged ≥ 30 years who were referred to the hospitals affiliated with the TUMS, Tehran province, Iran from 20, 2020, to March 10, 2021. Of 42,964 recorded patients, 22,162 patients were hospitalized (52.58%), and 20,802 patients (48.40%) were outpatient during the study period.

The study was approved by the National Institute for Medical Research Development, Tehran, Iran (IR.NIMAD.REC.1399.157) which act at the University/Regional level for biomedical research. The study was conducted in accordance with the guidelines and protocols of the Islamic Republic of Iran, Ministry of Health and Medical Education and with the Declaration of Helsinki. For the current study, the de-identified data were extracted from electronic health records, and consequently consent waiver was obtained from the National Institute for Medical Research Development ethic committee.

### Definitions

For the current study, the following symptoms were considered as respiratory symptoms: cough, respiratory distress, and chest pain. GI symptoms were defined as having any self-reported stomach pain, nausea, vomiting, diarrhea, anorexia and fever, muscle aches, loss of consciousness, convulsion, headache, dizziness, palsy of limbs, and organ damage. Eczema, loss of sense of smell, and loss of sense of taste were categorized as the other symptoms. We further categorized the study population into four groups depending on their disease symptoms: Group 1: patients with only GI symptoms, Group 2: patients with only respiratory symptoms, Group 3: patients with both symptoms: Group 4: patients with other symptoms.

Mortality (proportion of patients who died due to COVID-19 in hospital, and for outpatients who were cared at home, and health workers had followed them to recover via phone contact during disease. Therefore, the duration of the mortality study period was approximately 14 days after diagnosis) and hospitalization (proportion of patients who were hospitalized due to COVID-19) were defined as the study outcomes.

### Statistical analysis

Continuous variables were reported as mean ± standard deviations (SD), and qualitative variables were reported as frequency (%). The comparison of baseline characteristics of the four groups of participants including patients with gastrointestinal symptoms, patients with respiratory symptoms, patients with both symptoms, and patients with other symptoms. (Reference group) was performed using the ANOVA test for continuous variables and the Chi-square test for categorical variables.

Logistic regression was used to examine the relationship between symptoms subgroups with hospital admission and total mortality in three models; model 1, unadjusted (only symptoms subgroups); model 2, adjusted for baseline age, and sex, and model 3, adjusted for baseline age, sex, and other potential confounders including cancer, hypertension, coronary heart diseases, chronic liver diseases, and diabetes (showing statistical significance between four groups).

To show the robustness of our findings, we tested the interaction between the hospitalizations with our main exposures. Since the data analysis showed a significant interaction between hospitalizations and symptoms subgroups (*P* < 0.05), all analyses were repeated among those with and without hospitalizations.

A Kaplan–Meier survival curve was used to estimate the survival of the hospitalized patients according to each group. We repeated the analysis in hospitalized patients by using the Cox proportion hazard model. The level of significance was set at < 0.05 for all statistical analyses. All statistical analyses were done using STATA statistical package version 14 SE (StataCorp, TX, USA).

## Results

Overall, data of 42,964 confirmed cases of COVID-19 with a mean ± SD age of 51.36 ± 19.61 years were included in the analyses (53.19% were men). Overall, hospitalization and mortality rates were 52.88% (n = 22,162) and 8.83% (n = 3794), respectively. Mortality rates among hospitalized and non-hospitalized patient were 14.14% (n = 3134) and 3.13% (n = 652), respectively. Among hospitalized patients, the average days of hospitalization were reported to be 8.21 ± 9.2 days (Group 1: 7.3 ± 6.63 days; Group 2: 8.3 ± 8.3 days; Group 3: 7.53 ± 6.24 days; Group 4: 8.14 ± 9.5 days).

Among the total 42,964 cases of COVID-19, 1572 (3.70%) patients had GI symptoms in the absence of respiratory symptoms (Group1), 28,940 (67.35%) patients had respiratory symptoms in the absence of GI symptoms (Group 2), 2615 (6.10%) patients had both GI and respiratory symptoms (Group 3), and 9837 (22.89%) patients did not demonstrate any respiratory or GI symptoms (Group 4).

Table 1 shows the comparison of demographic characteristics of the patients’ subgroups with different symptoms. Generally, the highest and lowest mean of age was observed in group 3 (54.43 ± 19.28) and group 1 (47.50 ± 22.36), respectively (*P* = 0.001). Also, the prevalence of cigarette smoking (3.4%) and drug abuse (1.64%) in group 3 was higher than the other groups, and these differences were statistically significant (*P* = 0.001).

**Table 1:** Comparison demographic and health variables according to different symptoms subgroups

	Total (n = 42,964)	Group 1 (n = 1572)	Group 2 (n = 28,940)	Group 3 (n = 2615)	Group 4 (n = 9837)	*P* value
Age, years	51.36 ± 19.61	47.50 ± 22.36	52.42 ± 19.24	54.43 ± 19.28	48.02 ± 19.85	0.001
Gender						0.001
Women	20,110 (46.8)	813 (51.7)	13,359 (46.1)	1241 (47.46)	4697 (47.75)	
Men	22,854 (53.2)	759 (49.3)	15,581 (43.9)	1374 (52.54)	5140 (52.25)	
Cigarette smoking, yes	686(1.60)	26(1.65)	459(1.58)	89(3.40)	112(1.13)	0.001
Drug abuse, yes	351(0.82)	16(1.44)	227(0.78)	43(1.64)	65(0.66)	0.001

Continuous variables were reported as mean ± standard deviations (SD), and qualitative variables were reported as frequency (%). A one-way ANOVA test was used to explore differences between the symptoms subgroups. To comparison of qualitative variables between four groups, the Chi-square test was used

Group 1: Patients with Gastrointestinal symptoms; Group 2: Patients with respiratory symptoms; Group 3: Patients with both symptoms: Group 4: Patients with other symptoms

*Among hospitalized patients only (n = 22,162)

As shown in Table 2, cough and respiratory distress were more prevalent than other symptoms among all patients (49.48% and 42.63%, respectively). The highest frequency of different respiratory symptoms among patients with only respiratory symptoms (Group2) was cough 19,295 (66.67%) and respiratory distress 16,808 (58.07%), respectively. Among patients with respiratory and GI symptoms (Group 3), respiratory symptoms were illustrated as more prevalent than Group 2.

**Table 2:** Comparison some symptoms according to different symptoms subgroups

	Total (n = 42,964)	Group 1 (n = 1572)	Group 2 (n = 28,940)	Group 3 (n = 2615)	Group 4 (n = 9837)	*P* value
Respiratory symptoms						
Cough	21,260(49.48)	–	19,295(66.67)	1965(75.14)	–	0.001
Respiratory distress	18,317(42.63)	–	16,808(58.07)	1509(57.70)	–	0.001
Chest pain	1185(2.78)	–	644(2.22)	541(20.68)	–	0.001
Gastrointestinal symptoms						
Stomach ache	688(1.60)	296(18.82)	–	392(14.99)	–	0.001
Nausea	1781(4.14)	641(40.77)	–	1140(43.59)	–	0.001
Vomit	1051(2.44)	443(28.18)	–	608(23.25)	–	0.001
Diarrhea	1198(2.78)	502(31.93)	–	696(26.61)	–	0.001
Anorexia	1638(3.81)	506(32.18)	–	1132(43.28)	–	0.001
Other symptoms						
Fever	14,475(33.7)	514(32.7)	8588(29.67)	1171(44.78)	4202(42.71)	0.001
Muscular pain	15,896(37.0)	530(33.7)	9572(33.07)	1260(48.18)	4534(46.09)	0.001
Loss of consciousness	1188(2.8)	40(2.5)	718(2.48)	85(3.25)	345(3.50)	0.001
Convulsion	75(0.2)	13(0.82)	29(0.10)	3(0.11)	30(0.30)	0.001
Headache	1867(5.1)	193(12.27)	722(2.49)	507(19.38)	445(4.52)	0.001
Dizziness	787(2.15)	123(7.82)	249(0.86)	252(9.63)	163(1.65)	0.001
Palsy of the limb	172(0.5)	25(1.60)	62(0.21)	36(1.37)	49(0.49)	0.001
Organ lesion	50(0.15)	7(0.44)	11(0.03)	11(0.42)	21(0.21)	0.001
Eczema	33(0.09)	7(0.44)	7(0.02)	6(0.22)	13(0.13)	0.001
Loss sense of smell	1061(2.47)	4(0.002)	800(0.03)	257(0.10)	-	0.001
Loss sense of taste	727(1.70)	14(0.008)	443(0.02)	216(0.08)	54(0.005)	0.001

Continuous variables were reported as mean ± standard deviations (SD), and qualitative variables were reported as frequency (%). A one-way ANOVA test was used to explore differences between the symptoms subgroups. To comparison of qualitative variables between four groups, the Chi-square test was used

Group 1: Patients with Gastrointestinal symptoms; Group 2: Patients with respiratory symptoms; Group 3: Patients with both symptoms: Group 4: Patients with other symptoms

Regarding GI symptoms alone, the overall prevalence was reported less than respiratory symptoms, and the most prevalent GI symptoms in the whole population were nausea 1781 (4.14%), anorexia 1638 (3.81%), and diarrhea 1198 (2.78%), respectively. From the distribution point of view, Group 1 was the same as it was reported to be nausea 641 (40.77%), anorexia 506 (32.18%), diarrhea 502 (31.93%), vomiting 443 (28.18%), and stomachache 296 (18.82%). However, the distribution of GI symptoms was somehow different in patients who had both respiratory and GI symptoms (Group 3); the most common symptom among this group was nausea 1140 (43.59%), followed by anorexia 1132 (43.28%). Among other symptoms, muscular pain was the most prevalent symptom 15,896 (37.0%), followed by fever 14,475 (33.7%). More details for other symptoms in the four groups are shown in Table 2.

Regarding the underlying disease in Table 3, in total, coronary heart disease (9.21%) was the most prevalent comorbidity, followed by diabetes (8.10%), hypertension (7.52%), and cancer (1.89%); however, Group 3 showed the most prevalent underlying disease among other groups (*P* < 0.0001).

**Table 3:** Comparison of some comorbidities according to different symptoms subgroups

	Total (n = 42,964)	Group 1 (n = 1572)	Group 2 (n = 28,940)	Group 3 (n = 2615)	Group 4 (n = 9837)	*P* value
Comorbidities						
Cancer	814(1.89)	33(2.0)	518 (1.8)	93 (3.56)	170 (1.73)	0.001
Diabetes	3482(8.10)	184(11.7)	2351 (8.12)	434(16.60)	513 (5.22)	0.001
CHD	3958(9.21)	148(9.4)	2700 (9.32)	443 (16.94)	667 (6.78)	0.001
Hypertension	3232(7.52)	184(11.7)	2003 (6.92)	554 (21.19)	491 (4.99)	0.001
COPD/Asthma	450 (1.05)	9 (0.6)	364 (1.25)	34 (1.30)	43 (0.44)	0.001
Chronic lung diseases	577(1.34)	12(0.8)	419(1.44)	60 (2.29)	86 (0.87)	0.001
Chronic kidney diseases	689(1.60)	43(0.02)	441(0.02)	81(0.03)	124(0.01)	0.001
Chronic blood diseases	234(1.34)	9(0.01)	159(0.01)	20(0.01)	46(0.001)	0.32
HIV/AIDS	46(0.001)	1(0.001)	34(0.001)	1(0.001)	10(0.001)	0.62
Neurological disorders	288(0.006)	15(0.01)	162(0.01)	38(0.01)	73(0.01)	0.001

Qualitative variables were reported as frequency (%). To comparison of qualitative variables between four groups, the Chi-square test was used

Group 1: Patients with Gastrointestinal symptoms; Group 2: Patients with respiratory symptoms; Group 3: Patients with both symptoms: Group 4: Patients with other symptoms

CHD, coronary heart disease; COPD, chronic obstructive pulmonary disease

**Fig. 1:**
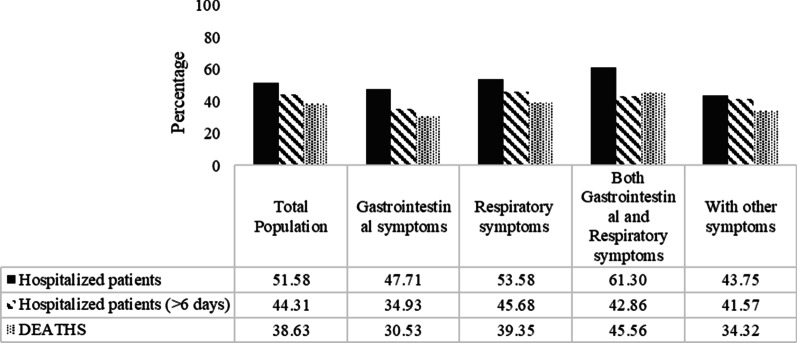
Distribution of patients’ situations (hospitalization, hospitalization > 6 days, and death according to different symptoms subgroups

**Fig. 2:**
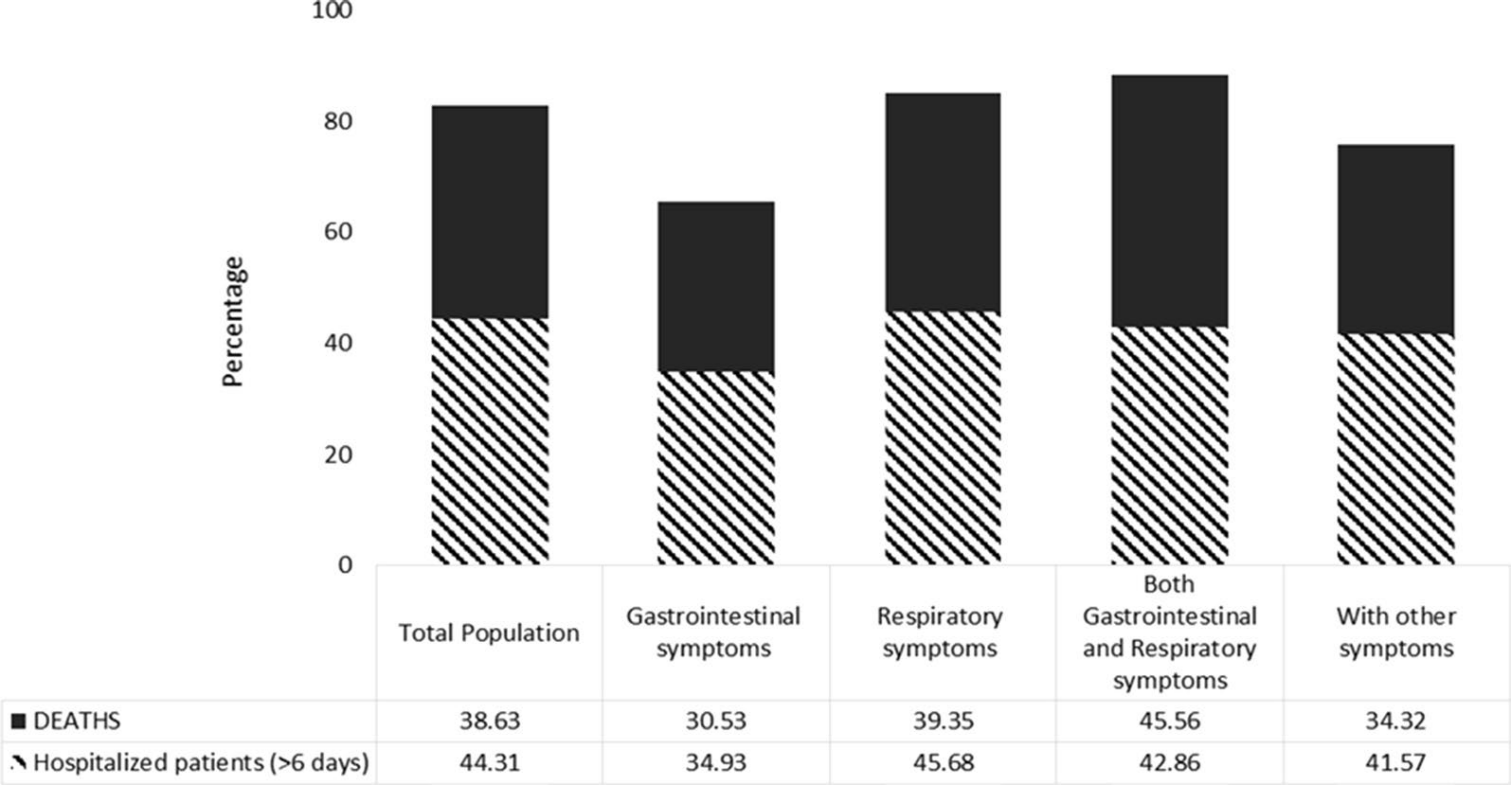
The proportion of death and hospitalization > 6 days in different symptoms subgroups

Table 4 shows the comparison of deaths, hospitalization, and prolong hospitalization (more than 5 days) according to different symptoms subgroups; 3, 794 (8.83%) were dead, and also 22, 162(52.88%) were hospitalized; however, 9,821(23.0%) patients had prolonged hospitalization. The most mortality rate was illustrated among the patients in Group 3 with 313 (11.97%) death, followed by Group 2 with 2,787(9.63%) (*P* = 0.001). The highest percentage of hospitalization was seen among the patients of Group 3 (61%) and Group 2 (53.6), respectively (*P* = 0.001). Group 2 (45.6%) and Group 3 (42.8%) had prolonged hospitalizations (*P* = 0.001) as well. More information about patients’ death and hospitalization according to different symptoms is depicted in Figs. 1 and 2.

**Table 4:** Comparison of deaths, hospitalization, and prolong hospitalization according to different symptoms subgroups

	Total (n = 42,964)	Group 1 (n = 1572)	Group 2 (n = 28,940)	Group 3 (n = 2615)	Group 4 (n = 9837)	*P* value
Death	3794(8.83)	80(5.08)	2787(9.63)	313(11.97)	614 (6.24)	0.001
Hospitalization	22,162(52.88)	750(47.7)	15,505(53.5)	1603(61)	4304(43.7)	0.001
Hospital stays^a^, day	8.21 ± 8.92	7.30 ± 6.63	8.30 ± 8.30	7.53 ± 6.24	8.14 ± 9.5	0.001

	Total (n = 22,162)	Group 1 (n = 750)	Group 2 (n = 15,505)	Group 3 (n = 1603)	Group 4 (n = 4304)	*P* value
Prolong hospitalization^a^ (≥ median 6 days)	9821(23.0)	262(34.9)	7083(45.6)	687(42.8)	1789(41.5)	0.001

Continuous variables were reported as mean ± standard deviations (SD), and qualitative variables were reported as frequency (%). To comparison of qualitative variables between four groups, the Chi-square test was used

Group 1: Patients with Gastrointestinal symptoms; Group 2: Patients with respiratory symptoms; Group 3: Patients with both symptoms: Group 4: Patients with other symptoms

^a^Was reported for hospitalized patients (n = 22,162)

The results of logistic regression analysis investigating the association between different symptoms groups as categorical variables with hospitalization and mortality as interested outcomes are presented in Table 5 (Groups 4 defined as the reference). The multivariable-adjusted odds ratios (OR; 95% confidence intervals: CI) for mortality were estimated to be 0.72 (0.56–0.92), 1.36 (1.24–1.50), and 1.52 (1.31–1.78) for Groups 1, 2, and 3, respectively. GI symptoms only significantly decreased the odds of mortality (as a preventive factor), and respiratory symptoms increased the odds of mortality (as a risk factor) compared to those with other symptoms. However, patients who had both symptoms (GI and respiratory) showed more odds of mortality in comparison with two other groups compared with those with other symptoms.

**Table 5:** Odds ratios (OR) for mortality and hospitalization according to different symptoms subgroups

	Model 1 OR (95% CI)	Model 2 OR (95% CI)	Model 3 OR (95% CI)
Outcome = death (n = 42,964, event = 37,94)			
With other symptoms	Reference	Reference	Reference
Gastro-intestinal symptoms	0.80(0.63–1.02)	**0.76(0.59–0.97)**	**0.72(0.56–0.92)**
Respiratory symptoms	**1.60(1.46–1.75)**	**1.35(1.23–1.49)**	**1.36(1.24–1.50)**
Both gastrointestinal and respiratory symptoms	**2.04(1.76–2.35)**	**1.60(1.38–1.87)**	**1.52(1.31–1.78)**
Outcome = hospitalization (n = 42,964, event = 22,162)			
With other symptoms	Reference	Reference	Reference
Gastro-intestinal symptoms	**1.18(1.06–1.31)**	**1.21(1.08–1.36)**	1.11(0.99–1.25)
Respiratory symptoms	**1.53(1.46–1.60)**	**1.40(1.33–1.47)**	**1.39(1.32–1.46)**
Both gastrointestinal and respiratory symptoms	**2.20(2.01–2.41)**	**1.97(1.79–2.17)**	**1.71(1.55–1.89)**

Model 1: symptoms subgroups

Model 2: Model 1 + age, sex

Model 3: Model 2 + Cancer, Hypertension, Coronary heart diseases, Chronic liver diseases, Diabetes, Asthma, Chronic lung diseases, Chronic neurological diseases

Significant values are bolded

Regarding hospitalization as an outcome, multivariable-adjusted OR (95% CI) were estimated at 1.11 (0.99–1.25), 1.39(1.32–1.46), and 1.71(1.55–1.89) calculated for subgroups 1, 2, and 3, respectively (Table 5).

Statistically, a significant interaction was observed between hospitalization and mortality. The associations between different symptoms and mortality were examined after stratifying hospitalizations and non-hospitalizations (Table 6). We found a significant association between GI symptoms as a preventive factor with mortality only among non-hospitalized patients (OR (95% CI); = 0.47(0.25–0.88)) but not among hospitalized patients (OR (95% CI); 0.79(0.59–1.04)).

**Table 6:** Odds ratios (OR) for mortality (outcome) based on hospitalized and no hospitalized patients according to different symptoms subgroups

	Model 1 OR (95% CI)	Model 2 OR (95% CI)	Model 3 OR (95% CI)
Non-hospitalized cases (n = 20,802, event = 652)			
With other symptoms	Reference	Reference	Reference
Gastro-intestinal symptoms	0.59(0.32–1.07)	0.55(0.30–1.01)	**0.47(0.25–0.88)**
Respiratory symptoms	**1.47(1.21–1.79)**	**1.28(1.05–1.57)**	**1.29(1.05–1.59)**
Both gastrointestinal and respiratory symptoms	**1.68(1.16–2.43)**	1.39(0.94–2.05)	1.19(0.80–1.78)
Hospitalized cases (n = 22,162, event = 3134)			
With other symptoms	Reference	Reference	Reference
Gastro-intestinal symptoms	0.80(0.63–1.04)	0.78(0.59–1.03)	0.79(0.59–1.04)
Respiratory symptoms	**1.41(1.27–1.57)**	**1.26(1.13–1.41)**	**1.27(1.14–1.42)**
Both gastrointestinal and respiratory symptoms	**1.66(1.42–2.96)**	**1.45(1.23–1.71)**	**1.46(1.23–1.73)**

Model 1: symptoms subgroups

Model 2: Model 1 + age, sex

Model 3: Model 2 + Cancer, Hypertension, Coronary heart diseases, Chronic liver diseases, Diabetes, Asthma, Chronic lung diseases, Chronic neurological diseases

Significant values are bolded

**Fig. 3:**
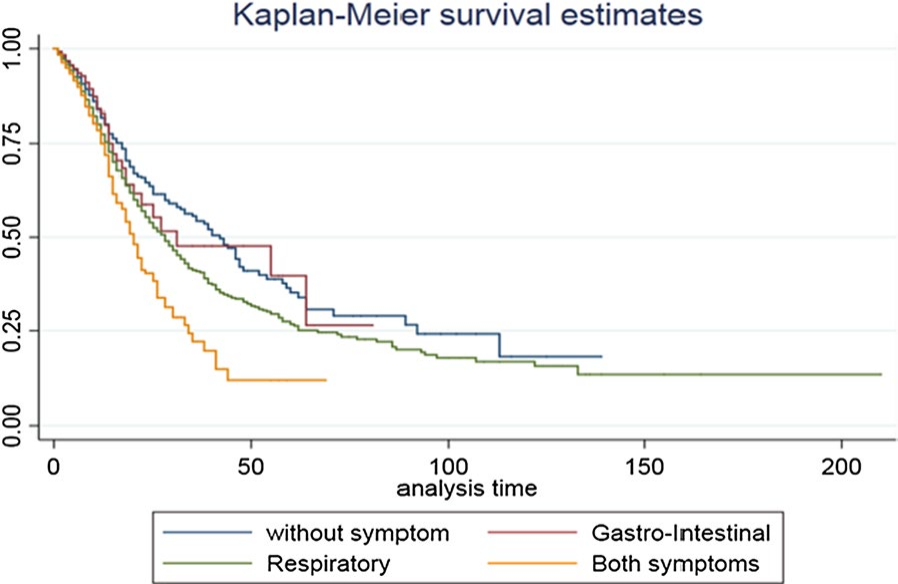
Kaplan Maier survival time for hospitalized patients (n = 22,162)

Finally, Table 7 shows the hospital length of stay among patients who were hospitalized. The hazard ratio in cox proportional hazard model was estimated for discharging from the hospital. The multivariable-adjusted hazard ratios for discharging from the hospital were estimated at (OR (95% CI); 1.21(1.11–1.32)), 0.92(0.88–0.95), 0.98(0.92–1.04) for Groups 1, 2, and 3, respectively. Figure 3 depicts the survival time of COVID-19 for hospitalized patients. As observed in Fig. 3, patients with GI symptoms had a better condition than those with respiratory symptoms.

**Table 7:** Hazard ratios (HR) for comparison hospital discharge (outcome) according to different symptoms subgroups

	Model 1 HR (95% CI)	Model 2 HR (95% CI)	Model 3 HR (95% CI)
*Hospitalized cases (n* = *22,162, event* = *3134)*			
With other symptoms	Reference	Reference	Reference
Gastrointestinal symptoms	**1.22(1.12–1.32)**	**1.20(1.10–1.30)**	**1.21(1.11–1.32)**
Respiratory symptoms	0.90(0.87–1.93)	**0.92(0.89–0.95)**	**0.92(0.88–0.95)**
Both gastrointestinal and respiratory symptoms	0.94(0.89–1.01)	0.97(0.91–1.03)	0.98(0.92–1.04)

Model 1: symptoms subgroups

Model 2: Model 1 + age, sex

Model 3: Model 2 + Cancer, Hypertension, Coronary heart diseases, Chronic liver diseases, Diabetes, Asthma, Chronic lung diseases, Chronic neurological diseases

Significant values are bolded

## Discussion

GI symptoms are commonly reported among COVID-19 patients. In our study, GI symptoms decreased the risk of COVID-19-related mortality (but not hospitalization) in the total population. This protective effect was found to be highly significant among non-hospitalized patients while not among hospitalized cases. We also observed that further respiratory symptoms increased the risk of mortality among hospitalized and non-hospitalized patients.

In this study, we found that 26.5% of the patients who were hospitalized with COVID-19 reported at least 1 GI symptom. Generally, the results of our investigation demonstrated that having GI symptoms in the absence of respiratory symptoms (Group 1) decreased the chance of mortality compared to Group 4 (crude odds ratio: 0.80). However, the differences were not statistically significant. Having respiratory symptoms in the absence of GI symptoms increased the chance of mortality compared to Group 4 (crude odds ratio: 1.60) which was statistically significant. And patients with both GI and respiratory symptoms had the highest chance of mortality (crude odds ratio:2.04) and hospitalization (crude odds ratio:2.20).

In agreement with our findings, a systematic review and meta-analysis found that approximately 10–12% of patients with SARS-CoV-2 infection had gastrointestinal symptoms [21]. The study by Pan et al. reported that 47% of patients had simultaneous respiratory and GI symptoms, 41% had only respiratory symptoms, and 3% had only GI symptoms [22].

The most common clinical symptoms among COVID-19 patients were cough, respiratory distress, body aches, and fever. In a systematic review study, the sequence of symptoms from the most to least common were fever, cough, sore throat, diarrhea, nausea, vomiting, and shortness of breath [23]. In our study, fever was less common than in other studies [24]. Multiple virus outbreaks and changes in patients’ clinical symptoms were observed over time. Multiple respiratory symptoms were higher among patients who had GI and respiratory symptoms simultaneously than patients who had only respiratory symptoms. The most common GI symptoms in our study were nausea (4.14%), anorexia (3.81%), and diarrhea (2.78%), respectively. In the study by Laszkowska et al., the most common GI symptoms included diarrhea (23.4%), nausea and vomiting (23.2%), and abdominal pain (11.9%). The age of patients with GI symptoms was lower than other patients, which was consistent with the results of other studies [25]. GI symptoms may be less reported due to more attention to respiratory symptoms, though.

The comorbidities in this study were cardiovascular disease, diabetes, and hypertension, respectively, agreeing with the findings of other studies [23, 26–29]. Some studies reported that the prevalence of chronic kidney disease was higher among patients with GI symptoms [30, 31]. In our study, the prevalence of chronic kidney disease in GI patients was similar to that in respiratory patients. The logistic regression analysis results also showed that GI symptoms could reduce the probability of mortality (as a preventive factor), and respiratory symptoms significantly increased the probability of mortality (as a risk factor). This finding is in line with the findings of other studies [32, 33].

Overall, 52.88% of PCR patients were hospitalized, 23% had long-term hospitalization (more than 5 days), and 8.83% of hospitalized patients died. In a study by Díaz et al., the percentage of hospitalization (16.5%) and mortality (1.1%) was lower than the percentages in our study [34]. The average number of hospitalization days was 8.21 ± 8.92 days, shorter than the day reported by Wang et al. [35] and Wen Zhao et al. [36]. The regular review of the length of stay of patients in the COVID-19 hospital reported varied from less than a week to nearly two months [37]. It has been reported that the likelihood of a lengthy hospital stay is associated with factors such as gender, having a fever as a symptom, and some diseases [38]. In the present study, patients’ clinical symptoms were associated with hospitalization and mortality. Patients who had GI and respiratory symptoms simultaneously had a higher severity of the disease than other patients, which led to an increase in hospitalizations (61%) and mortality (11.97%) in this group. Patients with only GI symptoms had a lower hospitalization rate (47.7%), long-term hospitalization (34.9%), and mortality (5.08%) than the other groups. In non-hospitalized patients, gastrointestinal symptoms were associated with death as a preventive factor. The study by Yang et al. showed that the length of hospital stay in patients with GI symptoms (12.13 ± 2.44 vs. 10.00 ± 2.13) was significantly longer than in patients with respiratory symptoms [39]. They had more than four times the chance of being hospitalized [31].

Many studies have reported that mortality in patients with GI symptoms is lower than in patients with respiratory symptoms [7, 40–43]. The results of our study revealed that patients with respiratory symptoms in the absence of GI symptoms had the highest percentage of long-term hospitalization (45.6%), and after Group 3, patients had the highest mortality (9.63%) and hospitalization percentages (53.5%). This can be due to the high prevalence of smoking and narcotics, the high severity of the disease in respiratory patients, and the lack of facilities, including the lack of mechanical ventilation and beds in the intensive care unit.

Of note, this study provides important insights into the association of hospitalization and mortality with the various symptoms of COVID-19 patients worldwide. One of the strengths of our research is the description of the characteristics of patients with COVID-19 in a large sample in Iran. Also, outliers and missing data in this study were very low. Although this study is the most extensive one comparing the symptoms of patients with COVID-19 with hospitalization and mortality, it is not without limitations. Because the criteria for hospitalization are diverse in different countries, it may be challenging to compare mortality in Iran with other countries. Some variables were recorded based on patients’ self-reports and are not verifiable. Also, the way patients’ information is reported in different hospitals is different, although there were instructions on recording data in the database. Databases contain only data recorded at the time of admission; information such as the exact time of onset of symptoms was not available to the researchers. Finally, it was impossible to follow up on discharged patients, so determining the mortality of patients with COVID-19 only included patients who died in the hospital.

In general, in this study, the percentage of hospitalization, duration of hospitalization, and mortality in four groups of patients with COVID-19 with different clinical symptoms were evaluated. The highest hospitalization and mortality rates were in patients with simultaneous respiratory and GI signs and patients with respiratory symptoms in the absence of gastrointestinal symptoms, respectively. The duration of hospitalization was longer in patients with respiratory symptoms in the absence of GI symptoms, patients with simultaneous respiratory and GI symptoms, patients with other symptoms, and GI patients without any respiratory symptoms.

## Conclusions

GI symptoms are commonly reported among COVID-19 patients. In our study, GI symptoms decreased the risk of COVID-19-related mortality (but not hospitalization) in the total population. This protective effect was found to be highly significant among non-hospitalized patients while not among hospitalized cases. Further respiratory symptoms increased the risk of mortality among hospitalized and non-hospitalized patients. More epidemiological studies are recommended to evaluate the association of GI and respiratory symptoms on hospitalization and mortality in COVID-19.

## Data Availability

The datasets used and/or analyzed during the current study are available from the corresponding author on reasonable request.

## Abbreviations

ARDS: Acute respiratory distress syndrome
ACE2: Angiotensin-converting enzyme receptors
TUMS: Tehran University of Medical Sciences and Health Services
MCMC: Medical Care Monitoring Center
GI: Gastrointestinal infectious
SD: Standard deviations
